# Automatic non-proliferative diabetic retinopathy screening system based on color fundus image

**DOI:** 10.1186/s12938-017-0414-z

**Published:** 2017-10-26

**Authors:** Zhitao Xiao, Xinpeng Zhang, Lei Geng, Fang Zhang, Jun Wu, Jun Tong, Philip O. Ogunbona, Chunyan Shan

**Affiliations:** 1 School of Electronics and Information Engineering, Tianjin Polytechnic University, No. 399 Binshui West Road, Nankai District, Tianjin, 300387 China; 2 Tianjin Key Laboratory of Optoelectronic Detection Technology and Systems, Tianjin, 300387 China; 3 School of Electrical, Computer and Telecommunications Engineering, University of Wollongong, Wollongong, NSW 2522 Australia; 4 Tianjin Medical University Metabolic Diseases Hospital, Tianjin, 300070 China

**Keywords:** Automatic screening system, Color fundus image, Early lesions, Non-proliferative diabetic retinopathy

## Abstract

**Background:**

Non-proliferative diabetic retinopathy is the early stage of diabetic retinopathy. Automatic detection of non-proliferative diabetic retinopathy is significant for clinical diagnosis, early screening and course progression of patients.

**Methods:**

This paper introduces the design and implementation of an automatic system for screening non-proliferative diabetic retinopathy based on color fundus images. Firstly, the fundus structures, including blood vessels, optic disc and macula, are extracted and located, respectively. In particular, a new optic disc localization method using parabolic fitting is proposed based on the physiological structure characteristics of optic disc and blood vessels. Then, early lesions, such as microaneurysms, hemorrhages and hard exudates, are detected based on their respective characteristics. An equivalent optical model simulating human eyes is designed based on the anatomical structure of retina. Main structures and early lesions are reconstructed in the 3D space for better visualization. Finally, the severity of each image is evaluated based on the international criteria of diabetic retinopathy.

**Results:**

The system has been tested on public databases and images from hospitals. Experimental results demonstrate that the proposed system achieves high accuracy for main structures and early lesions detection. The results of severity classification for non-proliferative diabetic retinopathy are also accurate and suitable.

**Conclusions:**

Our system can assist ophthalmologists for clinical diagnosis, automatic screening and course progression of patients.

## Background

Diabetes mellitus (DM) is a kind of serious metabolic disease characterized by the anomaly of insulin secretion. The chronic hyperglycemia of DM can cause complications of eyes, kidneys, nerves, heart, and blood vessels [1]. Diabetic retinopathy (DR) is one of the most severe complications of DM, which is the leading reason of blindness among adults aged 20–74 years [2]. DR can be classified into two stages: non-proliferative diabetic retinopathy (NPDR) and proliferative diabetic retinopathy (PDR). NPDR is characterized by structural damages to small fundus blood vessels, causing them to dilate, leak, or rupture. Visible retinal lesions include microaneurysms (MAs), hemorrhages, hard exudates, cotton-wool spots, intraretinal microvascular abnormalities (IRMA) and venous beading. MAs appear first in fundus. With the progression of NPDR, one or more dots or blot hemorrhages may develop successively. MAs, hemorrhages and hard exudates are early lesions of NPDR, which can be classified into mild, moderate, and severe stages depending on their severity [3]. NPDR is the early phase of DR, whose early detection is critical for diagnosis and treatment. Therefore, an automatic computer-aided NPDR screening system is indispensable.

**Fig. 1:**
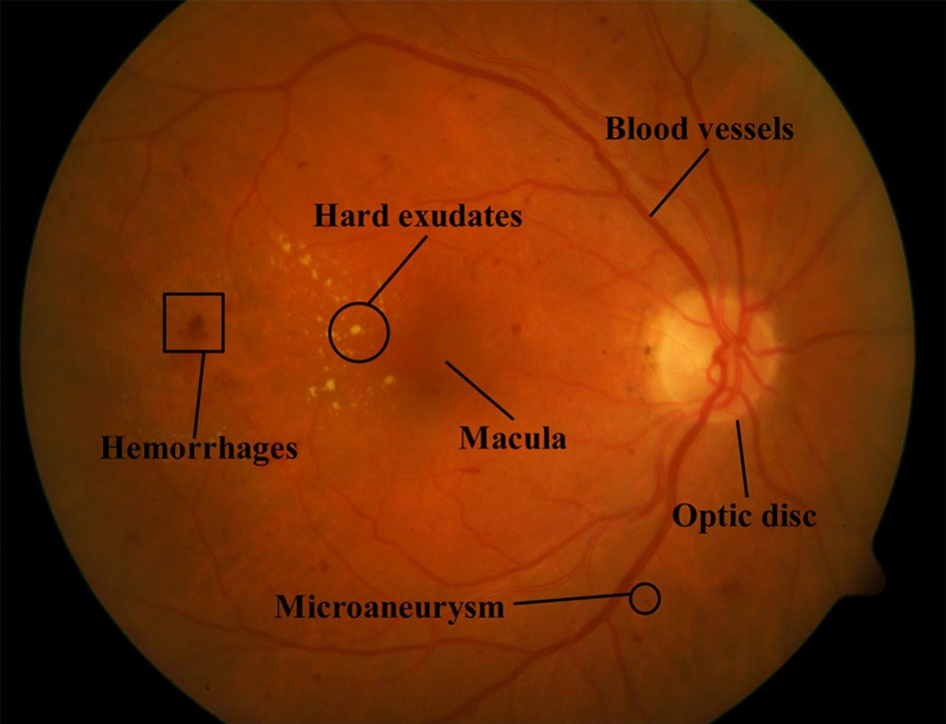
Illustration of main structures and early lesions in color fundus image

Common techniques for diagnosing early lesions include fluorescein angiography and color imaging. In contrast to fluorescein angiography, which may produce a number of side effects, color imaging is noninvasive and hence convenient for early lesions detection. Figure 1 depicts a typical color fundus image with main fundus structures and early lesions marked.

Several computer-aided NPDR screening systems based on color fundus images have been reported in the literature. Niemeijer et al. [4] proposed a scheme for detecting red lesions including MAs and hemorrhages based on pixel classification, which can detect large lesions without the need of knowledge about size and shape. García et al. [5] presented a method to detect red lesions. The features of red lesions and background were extracted for classification using multilayer perceptron classifier. Jaafar et al. [6] used classifiers to discriminate red lesions from background based on the features of fundus targets. Morphological processing was exploited to extract the candidates of red lesions and blood vessels. Then a set of features were extracted to classify the two kinds of targets. Although these methods can detect red lesions automatically and achieve high accuracy, they did not consider hard exudates. This is unsatisfactory for DR screening.

Sinthanayothin et al. [7] designed an automatic DR screening system to distinguish normal and abnormal fundus images. Hard exudates were detected using recursive region growing. MAs and hemorrhages were then recognized after removing blood vessels. Due to the poor performance of red lesions detection, only hard exudates were used for DR screening. Ravishankar et al. [8] developed another system to locate optic disc (OD) and detect lesions. Main blood vessels were segmented to locate OD. Hard exudates were then extracted after removing OD, and red lesions were detected by thresholding. Compared to [7], this system can predict the severity of DR based on the number and distribution of lesions. However, the method for locating macula utilizes the relative positioning of macula and OD. Inaccurate segmentation of OD will lead to the failure of macula localization. This method is also sensitive to the brightness of image and hemorrhages with large sizes. In addition, morphological filtering is unsuitable for detecting MAs with low contrast.

**Fig. 2:**
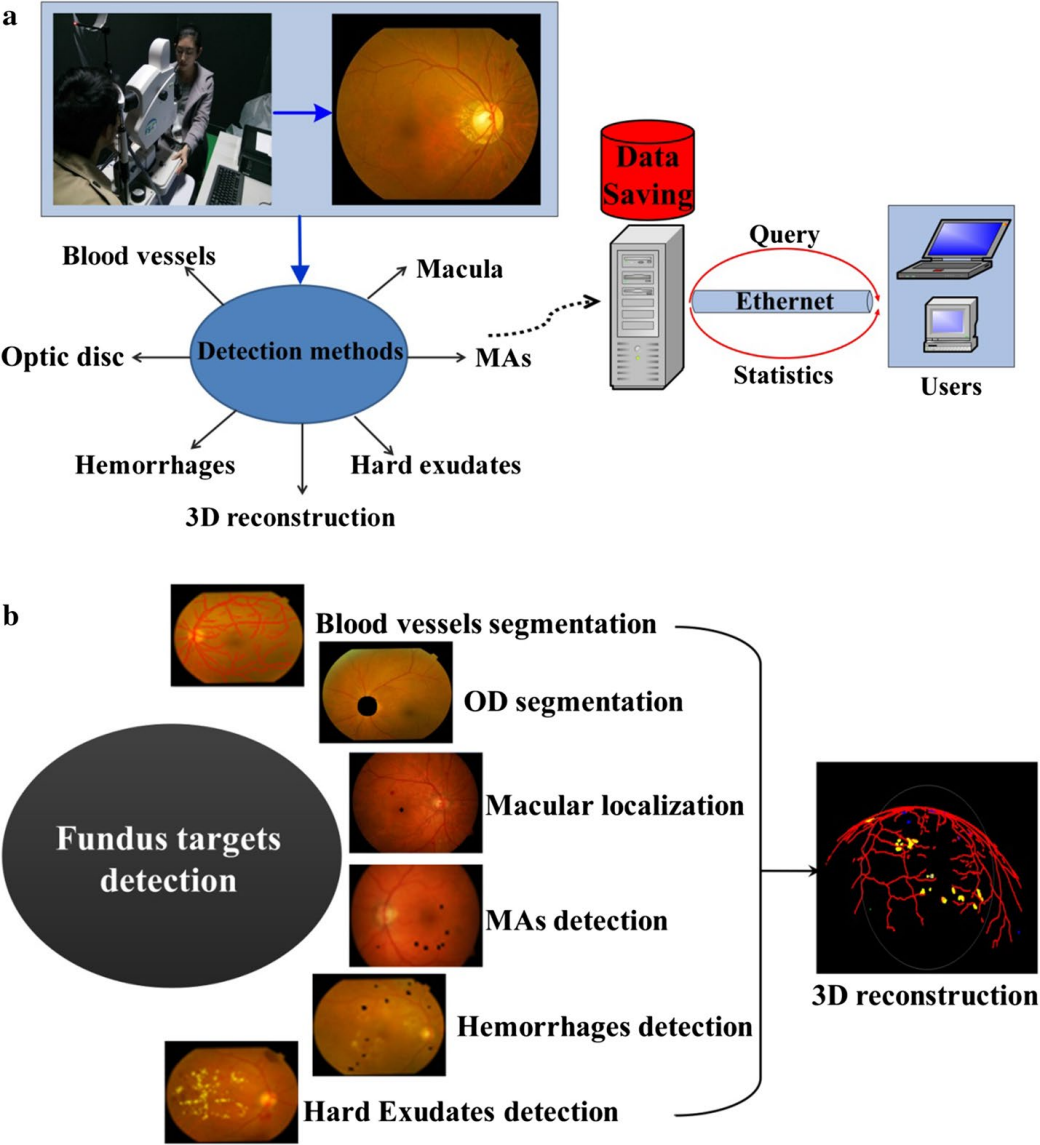
Automatic DR screening system. **a** Framework. **b** Fundus targets detection modules

This paper proposes a new automatic DR screening system exploiting the different features of main structures and early lesions. As described in Fig. 2a, the system realizes color imaging using fundus camera, fundus targets detection and saving, query and statistics of data. Figure 2b illustrates the detection modules. Main structures segmentation is used to distinguish and extract them and also to improve the performance of lesions detection. Since blood vessels exhibit linear structures with low contrast and MAs are weak targets affected by low contrast and noise, phase congruency (PC) is used to detect blood vessels and MAs [9] as PC is insensitive to brightness and noise. Based on the structural differences between OD and blood vessels, parabola fitting is used to locate the OD. The grayscale distribution of macula is hat-like, with minimum intensity at the macular center. Exploiting this feature, a macular detection method based on quadratic spline dyadic wavelet (QSDWT) [10] and gray contours is proposed. Based on the differences in the sizes and shapes of hemorrhages and hard exudates, a set of features are extracted to distinguish them using support vector machine (SVM). To overcome the limitation of traditional reconstruction methods for fundus images with front view, a 3D fundus reconstruction model based on the anatomical structures of human eyes is also proposed to better visualize the detection results.

This paper is organized as follows: “Background” section introduces backgrounds and aims of our system. “Proposed NPDR screening system” section details our system design, including techniques for blood vessel segmentation, optic disk segmentation, macular localization, and the detection of MAs, hemorrhages, and hard exudates. “Proposed NPDR screening system” section also discusses a 3D reconstruction scheme to better visualize the results. “Experimental results and discussion” section discusses the detection results and analyzes the performance. In “Conclusion” section concludes this paper.

## Proposed NPDR screening system

### Blood vessels segmentation based on PC

**Fig. 3:**
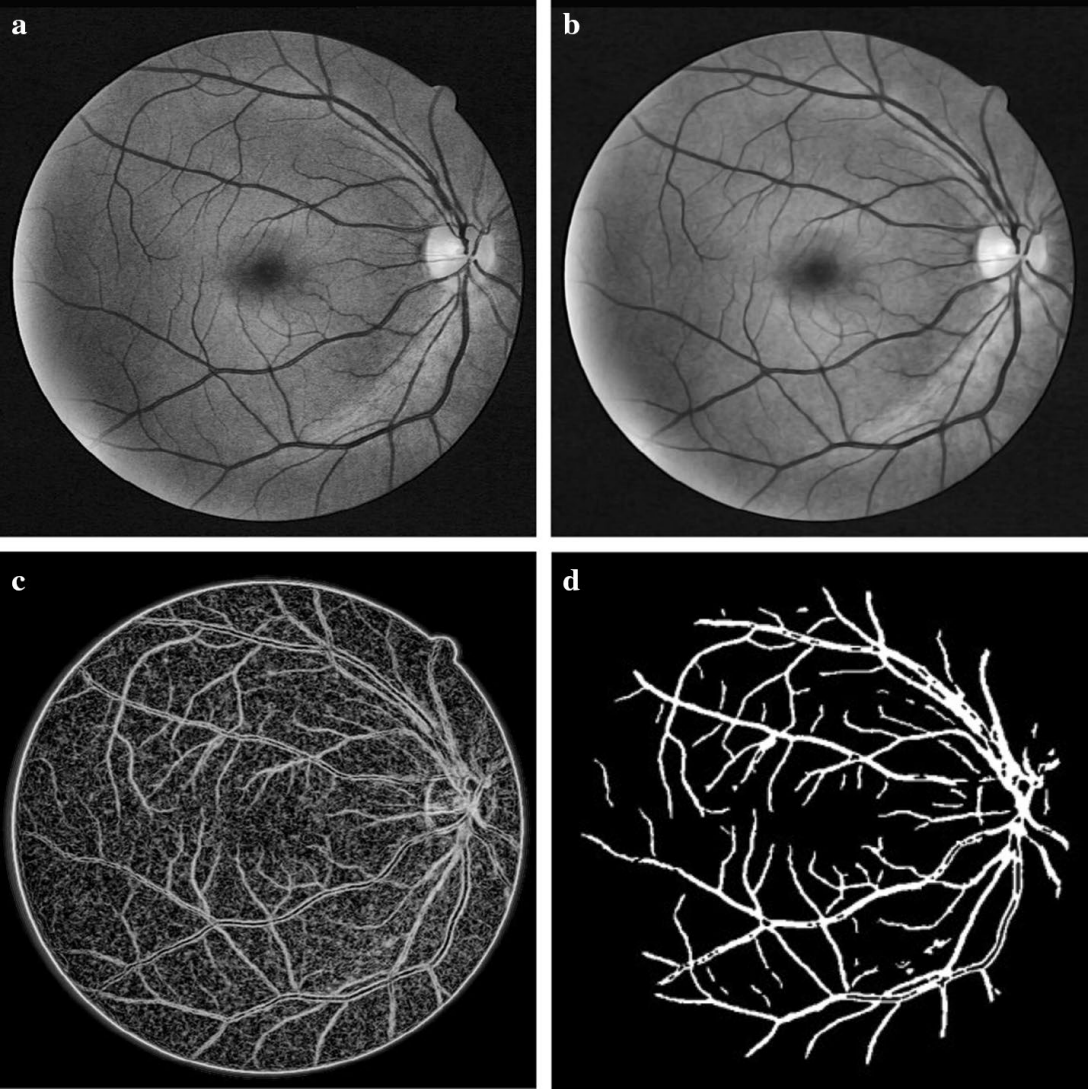
Segmentation results of blood vessels based on PC. **a** Contrast Enhancement using CLAHE. **b** Result of anisotropic coupled diffusion filtering. **c** Rough extraction of blood vessels. **d** Segmentation result after thresholding and area filtering

The green channel of the RGB model of the color fundus image has greater contrast and contains more information than the other two channels [11] and is thus selected for further processing. We adopt CLAHE to enhance the contrast. A retinal image is uniformly divided into non-overlapping blocks of equal sizes. Each block is then enhanced using histogram equalization, which can improve the local contrast, as shown in Fig. 3a. Since noises in the background can be enlarged, filtering is needed. Because of the directivity of blood vessels, anisotropic coupled diffusion filtering is chosen to reduce noise [12]. The anisotropic coupled diffusion filtering use the anisotropic coupled diffusion equations to filter along the edge direction, which improves the heat conduction equations. The anisotropic coupled diffusion equations are given by

1 $$\left\{ {\begin{array}{ll} {\partial_{t} u = g\left( {\left| {\nabla v} \right|} \right)\,\left| {\nabla u} \right|div\left( {\frac{\nabla u}{{\left| {\nabla u} \right|}}} \right) - \left[ {1 - g\left( {\left| {\nabla v} \right|} \right)} \right]\left( {u - M} \right)} \\ \partial_{t} v = a_{n} div\left( {\frac{\nabla v}{{\left| {\nabla v} \right|}}} \right) - b\left( {v - u} \right), \hfill \\ u\left( {x,y,0} \right) = M\left( {x,y} \right),\quad v\left( {x,y,0} \right) = M\left( {x,y} \right) \\ \end{array} } \right.$$

2 $$g\left( {\left| {\nabla v} \right|} \right) = \frac{1}{{1 + l\left| {\nabla v} \right|^{2} }}$$

3 $$a_{n} = \left\{ {\begin{array}{lll} {40\quad \quad \quad \quad\quad 1 \le n \le 9} \\ {a_{{n - 1}} - 0.7\;\;\;\;\;\;\;10 \le n \le 55} \\ {{{a_{{n - 1}} } / {2 \quad \quad \quad \; n \ge 55}}} \\ \end{array} } \right.,$$

where $u\left( {x,y,t} \right)$ is the filtering result; $v\left( {x,y,t} \right)$ is the correction factor to improve the ability of the filter. *u* and *v* are coupled with each other in Eq. (1). $\nabla u$ and $\nabla v$ are the gradient of *u* and *v* respectively; *t* can be considered as the number of iterations; *M* is the result after CLAHE; *b* is chosen as a fixed constant 0.02; *l* is chosen as a small constant and $a_{n}$ determines the degree of smoothness of filtering, which decreases with iterations *n*. Figure 3b illustrates the enhancement results after anisotropic coupled diffusion filtering. It can be seen that the noises are reduced effectively. Meanwhile, the details of the blood vessels can be retained.

PC employs several Log-Gabor filters with different orientations and scales, which is insensitive to brightness and noise. The PC model is defined as

4 $$PC\left( {m,n} \right) = \frac{{\sum {_{o} \sum {_{s} W_{o} \left\lfloor {E_{so} - T_{o} } \right\rfloor } } }}{{\sum {_{o} \sum {_{s} A_{so} \left( {m,n} \right) + \varepsilon } } }} ,$$

where $PC\left( {m,n} \right)$ denotes the response after PC at the point $\left( {m,n} \right)$; *o* and *s* are the numbers of orientations and scales, respectively; $W_{o}$ is the weight coefficient; $A_{so} (m,n)$ is the amplitude of filter with the *s*th scale and the *o*th orientation; $E_{so}$ and $T_{o}$ are the local energy and noise compensation factor, respectively. The small constant $\varepsilon \,\,{ = }\,\,0.00001$. The filtering orientation $o$, is chosen based on the physiological structure of the blood vessels in retina and $s$ determines the detection ability for Log-Gabor filter. More details including small blood vessels and noises of retinal image will be detected with a larger $s$. According to numerous experiments on different databases, $o = 8$ and $s = 3$ are chosen to construct the set of filters. To match the different widths of blood vessels, the factor of adjacent filters is chosen as 1.6. The response of PC is shown in Fig. 3c.

Pixel-level multiplication is proposed to eliminate the noises in the background. The result obtained by applying PC directly to the original green channel image is multiplied to the enhancement result. Then a binarization algorithm based on iterative thresholding and area filtering is used to remove small structures. The results of blood vessels segmentation are illustrated in Fig. 3d.

### Optic disc segmentation based on least square fitting and Hough transform

**Fig. 4:**
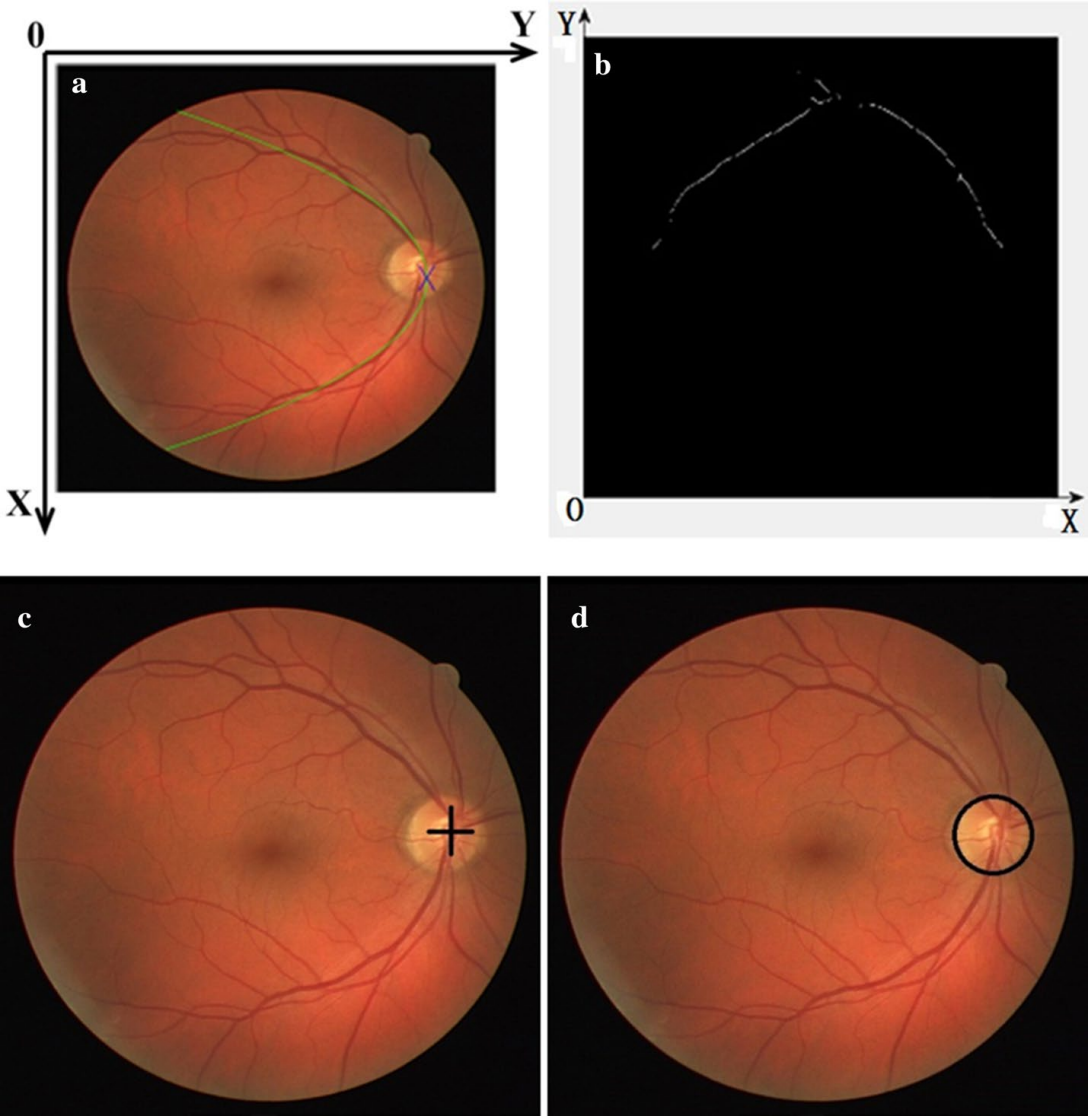
OD segmentation results using least square fitting and Hough transform. **a** The relative position between OD and main vein vessels. **b** Coordinate system construction. **c** Localization result using least square. **d** Segmentation result after Hough transform

OD is the starting point of all the blood vessels in retina. The main vein vessels can be modeled as a parabola. The center of OD is approximately located on the vertex of the parabola, as illustrated in Fig. 4a. Therefore, the skeleton line of the main vein vessels is extracted based on bottom-hat transform and iterative thresholding. The points of the skeleton line are mapped into the X–Y coordinate system, as shown in Fig. 4b. The coordinate set, $\left( {k_{i} ,l_{i} } \right){\kern 1pt} {\kern 1pt} {\kern 1pt} {\kern 1pt} {\kern 1pt} {\kern 1pt} \left( {1 \le i \le N} \right)$, is established to fit parabola. Here, *N* is the number of pixels on the skeleton line. A parabola can be defined as

5 $$f\left( k \right) = a_{0} k^{2} + a_{1} k + a_{2} .$$

To maintain the consistency of pixels, least square fitting is used to calculate the parabola parameters $a_{0} ,a_{1} ,a_{2}$ [13].

To get the position of the OD center, a region of interest (ROI) surrounding the vertex of parabola is first extracted. A circular sliding window whose diameter is 1/7 to 1/5 of the image width is designed to scan the ROI step by step. The luminance response of the center point is computed as

6 $$R\left( {u,v} \right) = \bar{I}_{u,v} \times \delta_{u,v} ,$$

where $\left( {u,v} \right)$ is the center of circular slide window; $\bar{I}_{u,v}$ is the mean of intensities in this window calculated from the A channel of the LAB color space, and $\delta_{u,v}$ is the variance of the intensities of the I channel of the HIS color space. The position of the maximum of $R\left( {u,v} \right)$ is considered as the center of OD, as described in Fig. 4c. OD is the brightest region with a circular shape. Hough transform is then used to segment OD [14], and the result is illustrated in Fig. 4d.

### Macular localization based on QSDWT and gray contours

Macular localization is significant for hemorrhages detection. Macula is the darkest region with a near-circular shape. The gray level distribution of the macula exhibits the inverse Gaussian behavior. To suppress the influences of lesions and uneven illumination, we propose to use QSDWT to extract the candidates of macular center and then establish the gray contours centered at the candidates to locate the macula accurately [15].

### MAs detection based on phase congruency

**Fig. 5:**
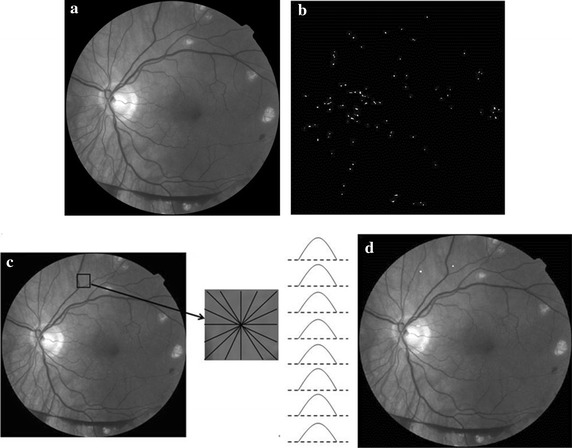
MAs detection results based on PC. **a** Green channel. **b** Response of PC. **c** Sample of cross-section profiles of an MA. **d** MA detection result after classification

MAs are small dark dots in the color fundus image, which are the first symptom of NPDR. It is difficult to detect MAs in the spatial domain. Phase information can describe the characteristics of edge, shape and texture. Lazar et al. indicates that MAs have a circular structure with a local maximum intensity from different directions after the operation of inversion in the green channel [16], as illustrated in Fig. 5c. Therefore, we propose a MA detection method based on PC and cross-section profiles [17]. A neighboring window of size *W* × *W* centered at a candidate is established. The gray values of pixels are scanned to paint cross-section profiles from 8 different directions (0°, 22°, 45°, 66°, 90°, 111°, 135°, 156°). *W* is empirically chosen as 15 in this system. Finally, the score based on the 7 feature points of cross-section profiles proposed by [16] is used to classify and the candidates whose scores within [20 30] are considered as true MAs, as shown in Fig. 5d.

### Hemorrhages detection based on k-means clustering and SVM

**Fig. 6:**
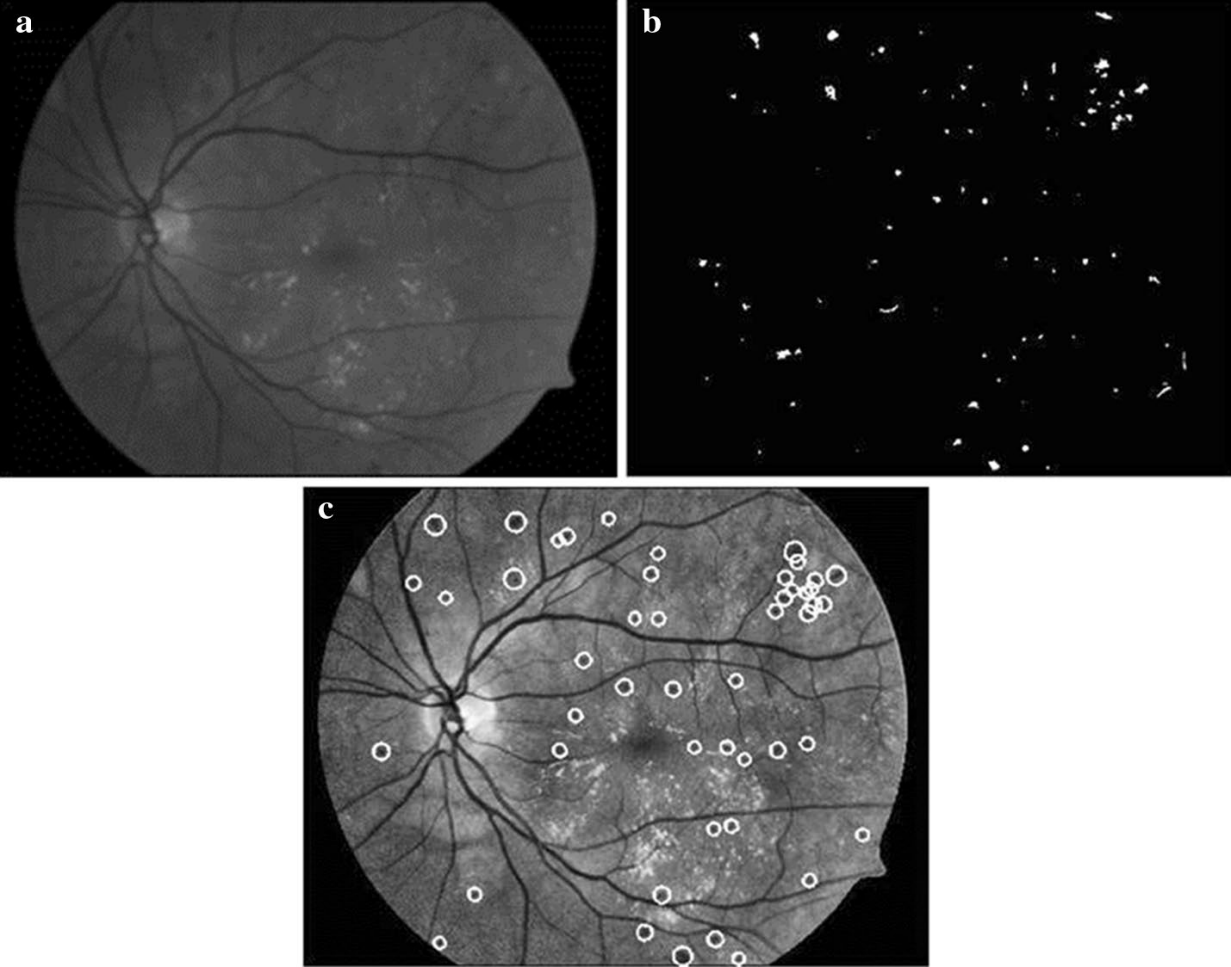
Hemorrhages detection results based on k-means clustering and SVM. **a** Enhancement result. **b** Candidate extraction result. **c** Classification result using SVM

Hemorrhages are red spots, which are important for predicting the severity of NPDR. We designed a method to detect dotted hemorrhages based on k-means clustering and SVM. Firstly, a median filter with size [80 × 80] is used to compute the background, which is then subtracted from the enhancement result using CLAHE, effectively reducing false negatives. Then, k-means clustering is used to distinguish hemorrhages and other structures. The function of k-means clustering is defined as

7 $$E = \sum\limits_{i = 1}^{K} {\sum\limits_{{x_{i} \in C_{i} }} {\left\| {x_{i} - c_{i} } \right\|^{2} } } ,$$

where $C_{i}$ is the *i*th class, $x_{i}$ is the gray values of a set of pixels classified as $C_{i}$ and $c_{i}$ is the mean of the gray values of pixels in $C_{i}$. The experimental results demonstrate that the number of clustering *K* = 5 is suitable to extract hemorrhages candidates. Because the gray values of blood vessels are similar to those of hemorrhages, the blood vessels are segmented using the method of “Blood vessels segmentation based on PC” section and removed to obtain the candidates of hemorrhages, as shown in Fig. 6b.

To compute the shapes of the candidates accurately, the method of region growing is applied to find the points whose features are similar to candidates in local regions. We select the centroid of each candidate as the start point. The criterion of region growing is given by

8 $$\left| {f\left( {x,y} \right) - m} \right| < T_{0} \quad and \quad f\left( {x,y} \right) < T$$

where *m* is the mean of gray value in the growing region, *T* is the threshold of each growing region calculated by Otsu, and $T_{0}$ is the cut-off condition of region growing, which is empirically chosen as 100. Finally, we designed a cascade classification scheme employing two SVM classifiers to reduce false positives due to blood vessels, backgrounds and residues after laser therapy. One is used to classify blood vessel fragments and hemorrhages based on shape features. The other is utilized to remove background noises using gray features [18]. The gray and structure features are described as follows.

1. Eccentricity;
2. Circularity: ${{p^{2} } \mathord{\left/ {\vphantom {{p^{2} } {4\pi a}}} \right. \kern-0pt} {4\pi a}}$, where $p$ is the number of pixels on the edge and $a$ is the number of pixels in each connected region;
3. Ratio between macro axis $l_{1}$ and minor axis $l_{2}$ of the ellipse having the same second-order central moment: ${{l_{1} } \mathord{\left/ {\vphantom {{l_{1} } {l_{2} }}} \right. \kern-0pt} {l_{2} }}$;
4. Mean of gray values for each candidate after region growing: $m_{{\text{in}}}$;
5. Mean of gray values in the neighborhood (the region excluding candidate): $m_{\text{out}}$;
6. Contrast: $$m_{\text{d}} = m_{{\text{out}}} - m_{{\text{in}}}$$.

The Standard Diabetic Retinopathy database (DIARETDB1) gives the ground truths of hemorrhages labeled by three ophthalmologists [20]. Each image of the DIARETDB1 database comes from a different patient. There are 39 images without hemorrhages and 50 images with hemorrhages. This database has comparable numbers of “positive” and “negative” samples and is used for training and testing the SVM. 393 positive samples and 384 negative samples are selected to train, as described in Table 1. Then two sets of feature vectors are obtained for classification. The classification results are shown in Fig. 6c.

**Table 1:** Training set constructed from DIARETDB1

Training samples	Number of samples
Positive	
Hemorrhages	393
Blood vessels	139
Negative	
Background	124
Residue after laser therapy	121

### Hard exudates detection

Hard exudates are bright lesions with well-defined edges but variable shapes. We adopt a method based on background estimation to detect hard exudates [19]. The bright objects including OD and hard exudates are acquired using background estimation, which is based on morphological reconstruction. Kirsch operation is utilized to detect the edges of bright objects. Then the candidates of hard exudates are extracted based on the thresholding of edge strength. After removing OD using the method of [14], several features including shape, gray and phase are extracted to classify hard exudates using SVM.

### 3D reconstruction based on anatomical structure of retina

**Fig. 7:**
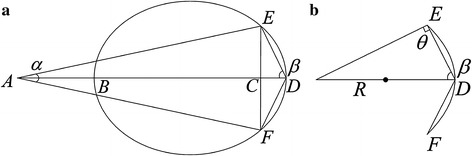
3D modeling of human eyes. **a** Optical model of human eyes. **b** Local fundus model with side view

The anatomical structure of retina includes the OD, macula and blood vessels network. The dioptric system of eye is composed of cornea, crystalline lens, vitreous body and aqueous humour. Fundus images are the posterior region with certain visual angles. Based on the optical properties of eyes, an equivalent optical model simulating human eyes is designed, as illustrated in Fig. 7a. In Fig. 7a, *A, B, C,* and *D* indicate the centers of the camera, the pupil, the fundus image and the posterior portion of fundus, respectively, and *E* and *F* are the upper and lower boundaries of the fundus image, respectively. $\overline{AB}$ and $a$ are, respectively, the working distance and field angle of the camera, $\overline{BD}$ is the length of the optical axis, and $\overline{EF}$ is the height of the fundus image. For the single fundus image, the coverage of the camera is region from E to D to F, which can be seen as a part of sphere, as described in Fig. 7b. The sphere is defined as

9 $$\left( {x - g} \right)^{2} + \left( {y - h} \right)^{2} + z^{2} = R^{2} ,$$

where $\left( {g,h} \right)$ is the center of the fundus image, $R$ is the radius, $\theta = 90^{ \circ }$,

10 $$R = {{\overline{ED} } \mathord{\left/ {\vphantom {{\overline{ED} } {(2\cos \beta }}} \right. \kern-0pt} {(2\cos \beta }}) ,$$

where

11 $$\overline{ED} = \left( {\overline{EC}^{2} + \overline{CD}^{2} } \right)^{{\frac{1}{2}}}$$

12 $$\overline{EC} = 0.5\overline{EF}$$

13 $$\overline{CD} = \overline{AB} + \overline{BD} - \overline{EC} \cot \left( {\frac{\alpha }{2}} \right)$$

In order to fully determine the geometric specifications, $\overline{BD}$ is measured by the optical machine Lenstar LS900,

14 $$\beta = \text{atan} \left( {\frac{{\overline{EC} }}{{\overline{CD} }}} \right) ,$$

**Fig. 8:**
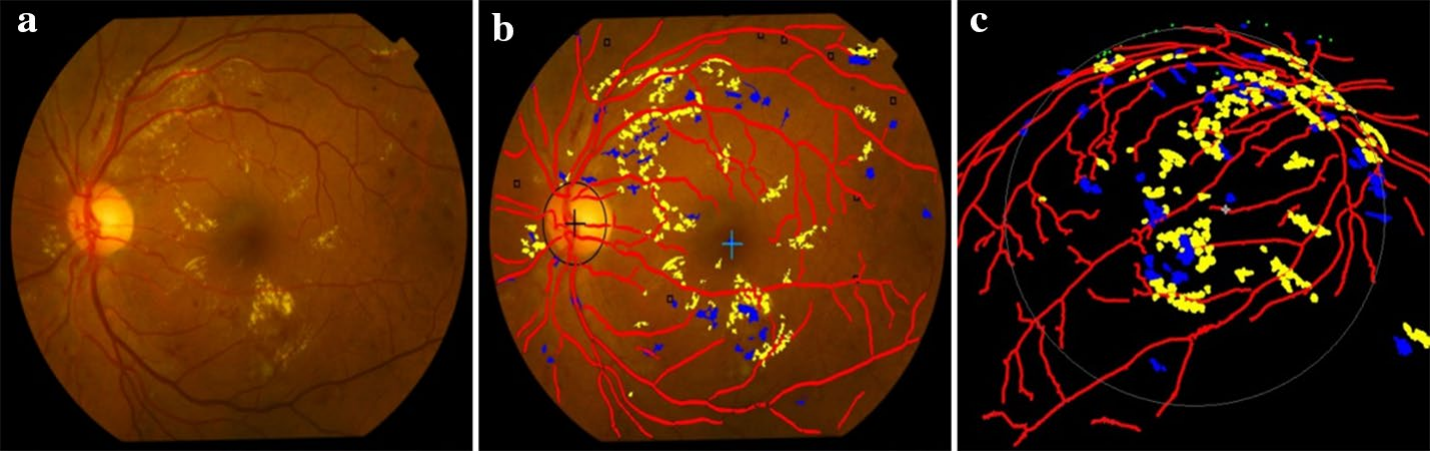
Detection results of the main fundus targets and 3D reconstruction based on human eyes structure. **a** Original color fundus image. **b** Detection results of the main structures and early lesions. **c** 3D reconstruction result of **b**

$\overline{AB}$, $\overline{EF}$ and $a$ are obtained directly from the working parameters of fundus camera. An example fundus image is shown in Fig. 8a where there exist MAs, dotted hemorrhages and other hard exudates. The detection results are superimposed on the original color fundus image, as shown in Fig. 8b. The red network shows the result of blood vessels segmentation. The centers of OD and macula are marked by “+”. Hemorrhages and hard exudates are detected and highlighted in blue and yellow colors, respectively. MAs are indicated as small squares. These detection results are expounded to the 3D space as shown in Fig. 8c.

## Experimental results and discussion

To evaluate the performance of the proposed DR screening system, a dataset containing 482 fundus images from five databases is constructed for experiments, including the DRIVE database [20], the Structured Analysis of the Retina (STARE) database [21], the Retinopathy Online Challenge (ROC) database [22], the Standard Diabetic Retinopathy database (DIARETDB) [23] and a database from Tianjin Medical University Metabolic Diseases Hospital (referred to as the Hospital hereafter). Specifically, DRIVE is a public database consisting of 40 fundus images for testing the blood vessels segmentation and macular localization. STARE, which was established by Goldbaum et al. contains 400 images for OD localization and segmentation. From STARE, we randomly selected 80 fundus images with different severities for testing. ROC was established for evaluating the performance of MA detection methods, with a training set consisting of 50 fundus images labeled by three ophthalmologists. DIARETDB includes two datasets DIARETDB0 and DIARETDB1 with 219 images. We used DIARETDB1, which includes 89 images with ground truths labeled by three ophthalmologists, to design detection methods for hemorrhages and hard exudates. We tested our detection methods on DIARETDB0 and also on The Hospital database containing 414 fundus images from different patients, which are classified and labeled by ophthalmologists. The Hospital database contains 414 fundus images from different patients, which are classified and labeled by ophthalmologists.

**Fig. 9:**
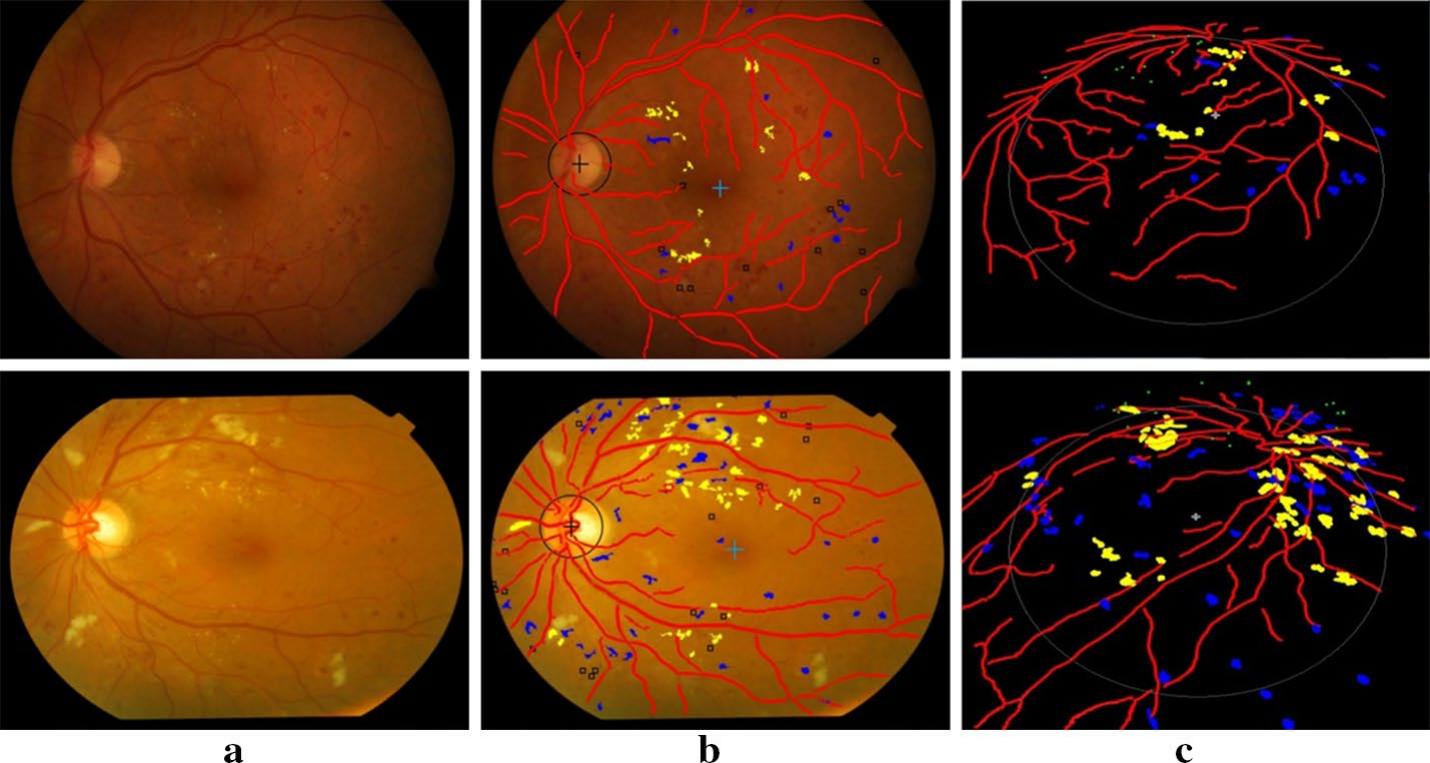
Example detection results of main structures and lesions using the proposed system. **a** Original color fundus images. **b** The detection results of (**a**). **c** 3D reconstruction results of (**b**)

Figure 9 presents examples of the detection results of the main fundus targets and 3D reconstruction achieved by using the proposed system. It can be seen that the OD and macula are located successfully with different brightness of the fundus images. The blood vessels network is fully extracted and the lesions are accurately detected with few false positives. One can also easily observe the location information of lesions from the 3D reconstruction results.

**Fig. 10:**
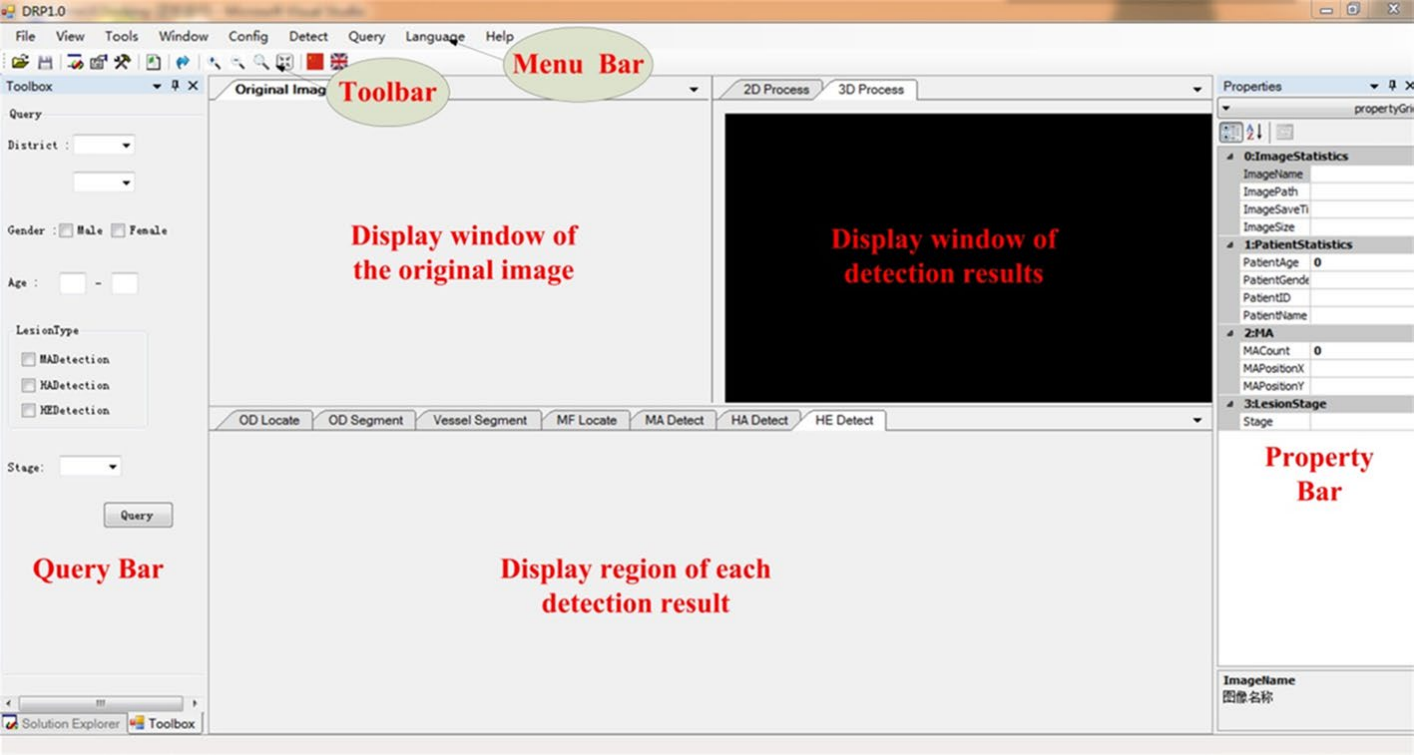
Main interface of automatic DR screening system

Figure 10 shows the main interface of the proposed DR screening system, which is designed in the VS2010 software environment. The original image is displayed in the left window. One or several detection results superimposed on the color images and 3D reconstruction are displayed for comparison. Every window can be displayed individually, which is convenient for ophthalmologists to observe. The detection results of the different types of targets can be displayed in the window below the original image according to the needs of the ophthalmologists. The menu bar provides functionalities image reading, targets detection, data saving, query, statistics and parameters setting. The toolbar consists of buttons for common functionality. The information of input images and predicting results are shown in the list of property. The region at the left-most of interface is set for simple query. The numbers of the three kinds of early lesions are calculated. Consequently, the severity of the detected image is predicted.

**Fig. 11:**
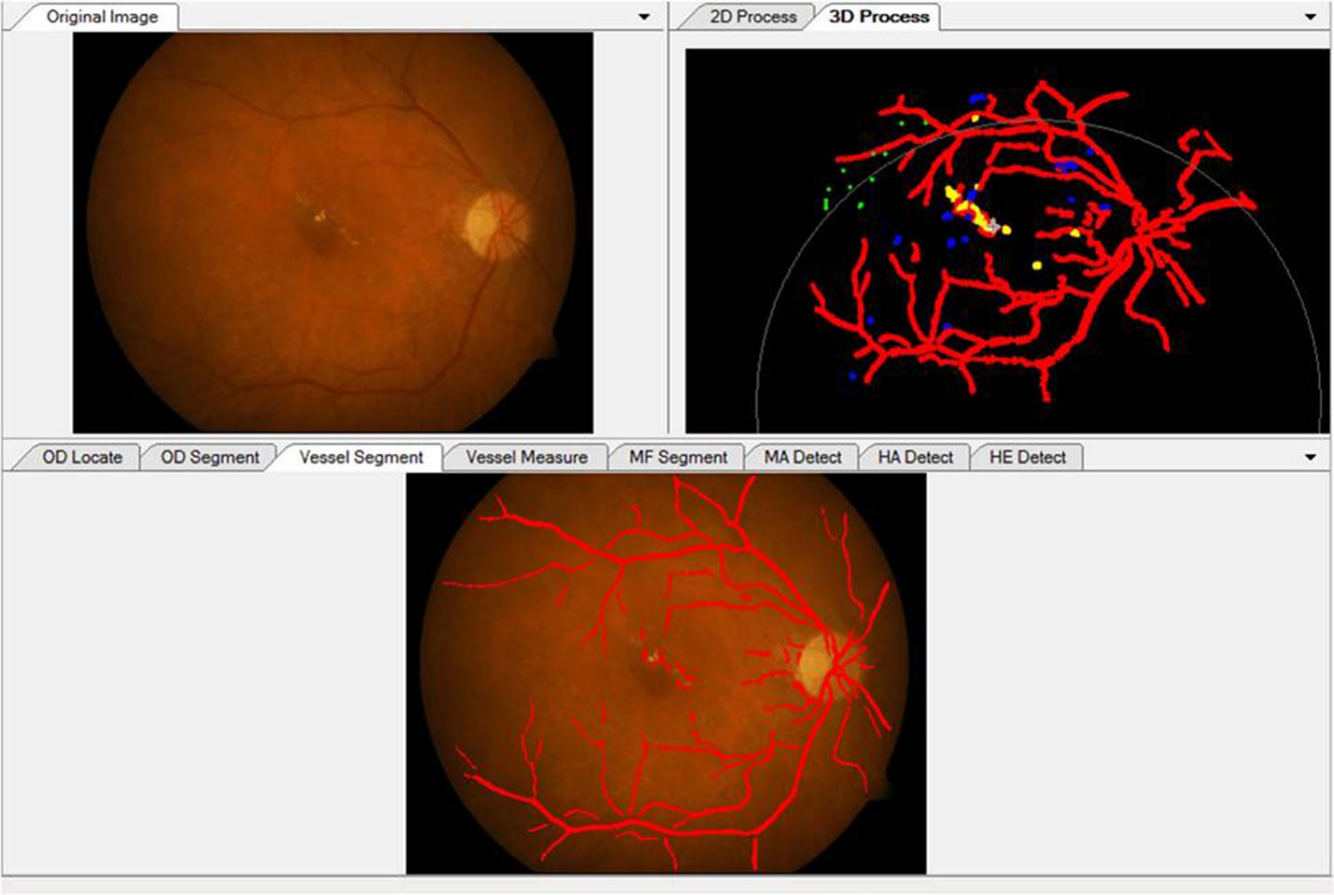
Sample of display widows for original image and detection results

**Fig. 12:**
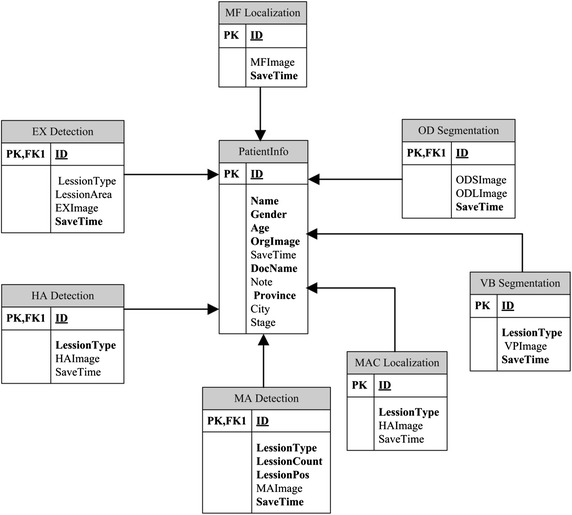
Information of data saving for patients and detection results

Figure 11 demonstrates the detection results displayed in the windows of the main interface. All the results can be moved out of the windows and observed separately at different sizes. We established a database for saving the information of patients and detection results, as illustrated in Fig. 12. Ophthalmologists can save and check the medical history and other information of patients in the database. In addition, users can retrieve the number of NPDR patients based on city, province, age, and gender.

Table 2 illustrates the sizes of retinal images in different databases. The resolution of images captured from Hospital is higher than other public retinal databases. In order to examine the influence of image resolution, we use the images with original sizes to test the performance of our methods.

**Table 2:** The resolutions of retinal images from different databases

Database	Image resolution
DRIVE	565 × 584
STARE	605 × 700
ROC	768 × 576, 1396 × 1392, 1386 × 1384, 1062 × 1061, 1058 × 1061, 1389 × 1383, 1381 × 1385
DIARETDB1	1500 × 1152
Hospital	2180 × 2000

Two different levels of evaluation, i.e., the image-level and lesion-level, are considered to evaluate the performance of our system. Measures of performance include sensitivity, specificity and accuracy. These can be computed as follows:

15 $${\text{Sensitivity}} = \frac{TP}{TP + FN}$$

16 $${\text{Specificity}} = \frac{TN}{TN + FP}$$

17 $${\text{Accuracy}} = \frac{TP + TN}{TN + FP + TP + FN}$$

where *TP* is true positive, *FP* is false positive, *TN* is true negative, and *FN* is false negative.

At the image level of evaluation, true positive (TP) implies that there is one or more lesions in the retinal image, and the method also detects the lesions but not necessarily all of them. True negative (TN) implies that there is no lesion in the retinal image, and the method also detects no lesion. False positive (FP) is the case when there is one or more lesions in the retinal image, but the method detects no lesion. Finally, false negative (FN) refers to the case where there is one or more lesions in the fundus image, but the method cannot detect any lesion.

Table 3 presents both the lesion and image level results. It can be seen that the image-level sensitivity and accuracy of early lesions of our method are higher than 90%, showing that our system can identify the presence of early lesions accurately. To assist ophthalmologists to diagnose in clinics, the system predicts the severity of NPDR based on the criteria of International Council of Ophthalmology. The severities of images from the Hospital are classified into mild and moderate. As shown in Table 4, our system achieves a 95% success rate of prediction. The detection results demonstrate that our methods are suitable for the images with different resolutions.

**Table 3:** Results of early lesions detection for all the databases

Early lesions	Sensitivity (%)	Specificity (%)	Accuracy (%)
MA (image level)	90	–	92
Hemorrhages (lesion level)	93	89	87
Hemorrhages (image level)	97	89	95
Hard exudates (lesion level)	84	94	–
Hard exudates (image level)	97	90	90

**Table 4:** Prediction results of severity of NPDR on hospital

Severity of NPDR	Number of images	Success rate (%)
Mild	65	95
Moderate	158	

Table 5 compares our method with two hemorrhages detection methods proposed by García et al. It can be seen that our method achieves higher sensitivity on lesion level than the method of [24] using four classifiers to detect hemorrhages. On the image level, the methods of [5] and [24] achieve 100% sensitivity, but their specificity and accuracy are unsatisfactory whereas our system achieves 80% sensitivity and 93% accuracy.

**Table 5:** Comparison of hemorrhages detection methods on DIARETDB1

Methods		Lesion level	Image level		
		Sensitivity (%)	Sensitivity (%)	Specificity (%)	Accuracy (%)
García et al. [5]		93	100	60	80
García et al. [24]	SVM	66	100	52	82
	MV	86	100	56	83
	MLP	85	100	52	82
	BRF	83	100	56	83
Our method		89	100	80	93

Blood vessels segmentation is significant for the precise detection of MAs and hemorrhages. Our method achieves 96% accuracy of blood vessels segmentation, as indicated in Table 6. OD and hard exudates are bright objects with similar characteristics. Accurate localization of OD can thus reduce the FPs of hard exudates detection. The performance of OD localization is shown in Table 7.

**Table 6:** Comparison of blood vessels segmentation methods on DRIVE

Methods	Accuracy (%)
Amin et al. [25]	92
Saffarzadeh et al. [26]	93
Vlachos et al. [27]	94
Our method	96

**Table 7:** Comparison of OD localization methods on STARE and DRIVE

Methods	Databases	Accuracy (%)
Haar et al. [28]	STARE	72
Youssif et al. [29]		88
Our method		94
Sagar et al. [30]	DRIVE	96
Welfer et al. [31]		100
Singh et al. [32]		95
Asim et al. [33]		100
Our method		100

To evaluate the performance of our method more accurately, the Hospital database is selected to detect fundus targets. The images are classified into three stages of severities, as described in Table 8.

**Table 8:** Classification of Hospital database by ophthalmologists

Severity	Kinds of early lesions	Number of images
Mild	MAs	65
Moderate	MAs, hemorrhages and hard exudates	155
Severe	MAs, hemorrhages and hard exudates	194

The numbers of early lesions in the severe fundus images are larger than those with moderate severity

Then, the main structures and early lesions are detected using the proposed DR screening system. The detection results are shown in Table 9. It is demonstrated that our system can segment and locate the main structures accurately. The main blood vessels network can be segmented except the thin ones. Based on the relationship between OD and main vein vessels, the center of OD is located using parabolic fitting. Because of the local shade in the fundus images, the accuracy of macula localization is reduced slightly. In addition, the proposed system achieves high sensitivity and accuracy in image level, which can detect the images with early lesions and ensure no false negative. Notably, this good performance demonstrates that our system can be applied for clinical diagnosis of NPDR and screening.

**Table 9:** Detection results of main structures and early lesions for the hospital database

Fundus targets	Sensitivity (%)	Specificity (%)	Accuracy (%)
Main structures			
Blood vessels	–	–	90
OD	–	–	100
Macula	–	–	96
Early lesions			
MAs (image level)	80	–	89
Hemorrhages (image level)	95	100	94
Hard exudates (image level)	100	89	94

## Conclusions

Based on the detection of main structures and early lesions from color fundus image, we designed an automatic NPDR screening system. The detected main structures include OD, blood vessels and macula, and early lesions include MAs, hemorrhages and hard exudates. In addition,the 3D optical model based on anatomical structure of human eyes is established to reconstruct the detection results in the 3D space. Five datasets are used to test and evaluate the performance of the system. The experimental results demonstrate that our system can detect early lesions accurately. The severity of NPDR can be predicted based on the detection results of early lesions. Our system can assist ophthalmologists for clinical diagnosis, automatic screening and course progression of patients.

## Abbreviations

DM: diabetes mellitus
DR: diabetic retinopathy
NPDR: non-proliferative diabetic retinopathy
PDR: proliferative diabetic retinopathy
MAs: microaneurysms
OD: optic disc
PC: phase congruency
QSDWT: quadratic spline dyadic wavelet
SVM: support vector machine
CLAHE: contrast limited adaptive histogram equalization
ROI: region of interest
STARE: structured Analysis of the Retina
ROC: Retinopathy Online Challenge
DIARETDB1: Standard Diabetic Retinopathy database
DIRVE: Digital Retinal Images for Vessel Extraction
